# Evaluation of genetic and phenotypic consistency of *Bacillus coagulans* MTCC 5856: a commercial probiotic strain

**DOI:** 10.1007/s11274-016-2027-2

**Published:** 2016-02-29

**Authors:** Muhammed Majeed, Kalyanam Nagabhushanam, Sankaran Natarajan, Arumugam Sivakumar, Talitha Eshuis-de Ruiter, Janine Booij-Veurink, Ynte P. de Vries, Furqan Ali

**Affiliations:** Sami Labs Limited, 19/1, 19/2, First Main, Second Phase, Peenya Industrial Area, Bangalore, 560 058 Karnataka India; Sabinsa Corporation, 20 Lake Drive, East Windsor, NJ 08520 USA; Life Sciences, FrieslandCampina Research, Bronland 20, 6708 WH Wageningen, The Netherlands

**Keywords:** *Bacillus coagulans* MTCC 5856, 16S rRNA, MLST, Genotypic fingerprinting, LactoSpore^®^

## Abstract

Commercial probiotics preparation containing *Bacillus coagulans* have been sold in the market for several decades. Due to its high intra-species genomic diversity, it is very likely that *B. coagulans* strain may alter in different ways over multiple years of production. Therefore, the present study focuses to evaluate the genetic consistency and probiotic potential of *B. coagulans* MTCC 5856. Phenotypic and genotypic techniques including biochemical profiling, 16S rRNA sequencing, GTG 5″, BOX PCR fingerprinting, and Multi-Locus-Sequence typing (MLST) were carried out to evaluate the identity and consistency of the *B. coagulans* MTCC 5856. Further, in vitro probiotic potential, safety and stability at ambient temperature conditions of *B. coagulans* MTCC 5856 were evaluated. All the samples were identified as *B. coagulans* by biochemical profiling and 16S rRNA sequencing. GTG 5″, BOX PCR fingerprints and MLST studies revealed that the same strain was present over 3 years of commercial production. *B. coagulans* MTCC 5856 showed resistance to gastric acid, bile salt and exhibited antimicrobial activity in in-vitro studies. Additionally, *B. coagulans* MTCC 5856 was found to be non-mutagenic, non-cytotoxic, negative for enterotoxin genes and stable at ambient temperature (25 ± 2 °C) for 36 months. The data of the study verified that the same strain of *B. coagulans* MTCC 5856 was present in commercial preparation over multiple years of production.

**Electronic supplementary material:**

The online version of this article (doi:10.1007/s11274-016-2027-2) contains supplementary material, which is available to authorized users.

## Introduction

The traditional use of probiotics in dairy products for human consumption has a long history in several parts of the world. As per FAO/WHO, probiotics are defined as “live microorganisms which, when administered in adequate amounts, confer a health benefit on the host” (FAO/WHO 2002). The most commonly used bacterial genera in probiotic preparations are *Lactobacillus, Bifidobacterium, Enterococcus, Bacillus* and *Streptococcus.* Some fungal strains belonging to *Saccharomyces* have also been used. Probiotics have been shown to be effective in varied clinical conditions—ranging from infantile diarrhoea, necrotizing enterocolitis, antibiotic-associated diarrhoea, relapsing *Clostridium difficile* colitis, *Helicobacter pylori* infections, inflammatory bowel and female urogenital infection (Gill and Prasad 2008; Shida and Nomoto 2013). There are number of probiotics strains used in dietary supplements and foods worldwide. However, the contents of commercial probiotics intended for both human and animal use are often not accurately represented on their labels. Several studies conducted independently revealed that a large percentage of products did not contain the specified organisms, contained other species of organisms, or did not contain the stated numbers of organisms (Hamilton-Miller and Shah 2002; Weese 2002; Hughes and Hillier 1990; Gilliland et al. 1984; Canganella et al. 1997). A survey on probiotics sold in Italy revealed that many of the commercial probiotic products do not comply with what their labels claim and consequently with what the Italian guidelines suggest. Notably, *B. coagulans* was absent in 5 of 17 preparations that were presumed to contain *B. coagulans* (“*L. sporogenes*”) according to the label, and also only in 8 of 17 preparations with *B. coagulans* the authors were able to find living *B. coagulans* cells/spores (Aureli et al. 2010). Hence, there is a need to identify the specified organism claimed to be present in commercial probiotic preparations.

*Bacillus coagulans* is a very heterogeneous species with a high intra-species genomic diversity (De Clerck et al. 2004). This was confirmed by Patel et al. (2006), who isolated a number of thermophilic acid tolerant bacteria most of which belonged to the species *B. coagulans*. Notably, these authors also found that *B. coagulans* apparently has several different type strains, meaning that the “type strain” has a different 16S rRNA sequence when ordered from different culture collections. *B. coagulans* genome contains a very large number of insertion sequence (IS) elements, which leads to major difficulties in assembly of the sequence and to “reshuffling” of the genome during growth/stress, potentially causing variability between different batches of production over a period of time. Hence, it is essential to evaluate the phenotypic and genotypic consistency of probiotic ingredients containing *B. coagulans*.

LactoSpore^®^ is a commercial probiotic preparation which contains the spores of *B. coagulans* MTCC 5856 (earlier known as *Lactobacillus sporogenes).* Commercial preparations of *B. coagulans* in powder, tablet and capsule forms have been successfully used in several human studies for the treatment of gastrointestinal disorders, vaginal infections, hypercholesterolemia, lactose intolerance, hepatic coma and as an adjuvant to antibiotic therapy (Majeed and Prakash 1998; Anonymous 2002; Jurenka 2012). The primary aim of this study was to assess the genetic consistency of *B. coagulans* MTCC 5856 strain over 3 years of commercial production by biochemical profiling and advanced molecular typing techniques. *B. coagulans* MTCC 5856 has been established safe and effective for human use over the last two decades (Majeed and Prakash 1998). However, it is essential to evaluate probiotic potential, safety and stability of *B. coagulans* MTCC 5856 as these properties may also be the indicators of genetic stability.

## Materials and methods

### Bacterial strains

The bacteria used in this study included *Staphylococcus aureus* ATCC 29213, *Staphylococcus epidermidis* ATCC 14990, *Streptococcus mutans* MTCC 1943, *Propionibacterium acnes* ATCC 11827, *Bacillus cereus* ATCC 14579, *Pseudomonas aeruginosa* ATCC 9027, *Escherichia coli* ATCC 25922, *Salmonella abony* NCIM 2257, *Micrococcus luteus* NCIM 2169 and *Salmonella typhimurium* histidine auxotrophs (TA98, TA100, TA102, TA1535 and TA1537). The reference strains were purchased from ATCC (American Type Culture Collection, Manassas, VA, USA) and MTCC (Microbial Type Culture Collection and Gene Bank, Chandigarh, INDIA). Cultures were maintained on agar plate and stocks were preserved in glycerol (15 % v/v) and stored at −80 °C.

### Genetic consistency of *B. coagulans* MTCC 5856

#### Test samples

Five production lots of *B. coagulans* MTCC 5856 (bearing internal reference number *B. coagulans* SBC37-01) were randomly selected for the study i.e. two samples from the years 2008 (G80241 and G80270) and 2009 (G90236 and G90467) and one from the year 2010 (G100241). Original culture was used as reference sample in the study.

#### Physiological profiling

Samples were subjected to microscopy (Lomo, St. Petersburg, Russia) and each sample was grown on glucose yeast extract agar (GYEA, HiMedia, India). From each sample three independent colonies were picked and subjected to biochemical characterization based on sugar fermentation pattern in basal broth medium as per the standard method described earlier (Kämpfer et al. 1991). Original culture (*B. coagulans* MTCC 5856) was used as reference sample in the study.

#### 16S rDNA sequencing

Genomic DNA of the *B. coagulans* MTCC 5856 was prepared as previously described by Marmur (1961). A fragment of the 16S rDNA gene was sequenced using an ABI 3100 automated DNA sequencer as described earlier (Heyrman and Swings 2001). The sequencing primers used were 5′-GAG TTT GAT CCT GGC TCA-3′ (forward primer, corresponding to positions 9–27 in *E. coli* numbering) and 5′-ACG GCT ACC TTG TTA CGA CTT-3′ (reverse, 1498–1477). The amplified DNA fragment of approximately 1.5 kb separated on a 1 % agarose gel and purified by using Qiagen spin columns. The purified fragment was used directly for DNA sequencing. This sequence was used in a BLAST search (http://blast.ncbi.nlm.nih.gov/Blast.cgi).

#### GTG 5″ and BOX PCR fingerprinting

Two variations of rep-PCR genomic fingerprinting were performed using the BOX and (GTG)5 primers. Colonies from *B. coagulans* MTCC 5856 production batch samples from three different years (2008, 2009, and 2010) were isolated. Three separate colonies from each sample were selected followed by total DNA isolation and then were subjected to GTG 5″ and BOX PCR fingerprinting. BOX PCR fingerprinting of the five *B. coagulans* MTCC 5856 production batch samples was performed using repetitive intergenic DNA sequences (rep-PCR) with the BOX AlR [5′-CTACGGCAAGGCGACGCTGACG-3′] primer. Optimal PCR program for the BOX PCR fingerprinting was used as previously described by Louws et al. (1994). (GTG)5 fingerprinting of the five *B. coagulans* MTCC 5856 production batch samples was performed using repetitive intergenic DNA sequences (rep-PCR) with (GTG)5 primer (5-GTGGTGGTGGTGGTG-3). The optimal PCR and the amplification cycling conditions for the rep-PCR were followed as described earlier by Versalovic et al. (1994). Products were separated by electrophoresis in 2 % agarose in 1× TBE buffer for 3.5 h at 65 V, and visualized by staining with ethidium bromide (0.5 mg mL^−1^) under ultraviolet light, followed by digital image capturing using a CCD camera. The reproducibility of the fingerprint profiles obtained was assessed in at least three separate experiments. The resulting fingerprints were analyzed by the BioNumerics V3.0 software package (Applied Maths, Belgium). Similarities of digitized profiles were calculated using the Pearson correlation method and an average linkage (UPGMA) dendrogram was obtained.

#### Multi-Locus-Sequence typing (MLST)

Four different samples (three production batches, G80241, G90236, G90467 and one original culture) of *B. coagulans* MTCC 5856 were characterized by the Multilocus Sequence typing as described by Priest et al. (2004) with minor modifications (Hoffmaster et al. 2008). Six household genes were targeted (*pta*, *tpi*, *glpF*, *ilvD*, *ldh*, and *pur*). Specific primers suitable for *B. coagulans* were developed and high fidelity PCR was performed. Amplification products of the correct size and similar concentrations, as judged by visual inspection of agarose gels, were obtained from all the samples and were then purified by using a QiaAmp PCR purification kit (Qiagen Inc., Valencia, CA) according to the instructions of the manufacturer. The purified fragment was used directly for DNA sequencing with an Applied Biosystems model 3100 automated DNA sequencing system.

### In vitro probiotic evaluation of *B. coagulans* MTCC 5856

#### Resistance to gastric acid

The survival of *B. coagulans* MTCC 5856 spores was studied by the addition of 1 mL of the suspension into a series of 9 mL of sterile phosphate-buffered saline (PBS) at pH 1.5, 3, 4, 5, 6, 7 and 8 (adjusted using 1 N NaOH and 1 N HCl). The incubation temperature was maintained at 37 °C and 1 mL sample was taken at 0, 0.5, 1.0, 2.0, 3.0 and 4.0 h. After incubation, serial dilution was done in sterile saline (0.89 % w/v) and the viable count was enumerated by plating on glucose yeast extract agar (HiMedia). Experiments were performed in triplicate at two different occasions.

#### Bile tolerance test

Bile tolerance of *B. coagulans* MTCC 5856 cells was determined by the method described earlier (Gilliland et al. 1984; Hyronimus et al. 2000). Briefly, overnight grown *B. coagulans* MTCC 5856 in MRS broth (20 μL corresponding to 2 × 10^6^ cfu mL^−1^) was spotted onto MRS agar plates containing oxgall bile (0.1–1 % w/v) (HiMedia, Mumbai, India). Plates were incubated at 37 °C for 5 days. The minimal inhibitory concentration (MIC) of bile for *B. coagulans* MTCC 5856 was determined as the lowest concentration totally inhibiting the growth of spots as judged from visual examination of spots. MRS broth (HiMedia) was inoculated with approximately 10^6^ cfu mL^−1^ of *B. coagulans* MTCC 5856 overnight grown culture and then supplemented with 0.3 (w/v) and 0.5 % (w/v) oxgall. Samples were incubated for 24 h at 37 °C with shaking at 120 rpm. Growth in control (no bile) and test cultures (0.3 and 0.5 % oxgall) was monitored hourly by measuring absorbance at 600 nm using spectrophotometer (Shimadzu Corporation, Kyoto, Japan). In same set of experiment, the viable count of *B. coagulans* MTCC 5856 was determined in triplicate on glucose yeast extract agar (HiMedia) at 0 and 24 h by pour plate method (Majeed et al. 2016).

#### Antimicrobial activity against human pathogens

The anti-microbial activity of *B. coagulans* MTCC 5856 was determined by growing in MRS media. A loopful of overnight grown culture was added to MRS media and incubated for 24 h at 37 °C with 120 rpm. After 24 h, the culture was centrifuged (10, 000×*g*) to remove the cells and the supernatant was collected, concentrated tenfold by lyophilization and filter-sterilized through a 0.22 micron filter (Sartorius, India). The antimicrobial activity was performed by a well diffusion assay as previously described with minor modifications (Cintas et al. 1995). Briefly, a 5 mL lawn of soft (0.7 % agar) glucose yeast extract (HiMedia, India), containing 10^6^ cfu mL^−1^) of the indicator strains (*S. aureus* ATCC 29213, *S. epidermidis* ATCC 14990, *S. mutans* MTCC 1943, *P. acnes* ATCC 11827, *B. cereus* ATCC 14579, *P. aeruginosa* ATCC 9027, *E. coli* ATCC 25922, *S. abony* NCIM 2257 and *M. luteus* NCIM 2169) was poured on top of an enriched hard (1.5 % agar) tryptic soya agar (HiMedia, India). Concentrated supernatant (50 µL) was added to 6-mm wells punched in the solidified bi-layer agar. Plates were kept in the refrigerator (4 ± 2 °C) for 5 h to allow the sample to diffuse into the agar and subsequently incubated at 37 °C for 18–20 h. After incubation, the zone of inhibition was measured and recorded in mm.

#### Production of lactic acid

A loopful of an overnight grown culture of *B. coagulans* MTCC 5856 was added to glucose yeast extract broth (HiMedia) and incubated at 37 °C for 18 h with 120 rpm. After incubation, the broth was filtered through 0.22 micron (Sartorius, India) and analyzed for lactic acid content by using Megazyme kit (K-DLATE 10/04) as per instructions (Megazyme International Ireland, IDA Business Park, Wicklow, Ireland). GYE media was taken as blank in the assay. The optical rotation of lactic acid produced by *B. coagulans* MTCC 5856 was confirmed by Polarimetric method (Kahya et al. 2001). Above filtered broth (50 mL) of the overnight grown *B. coagulans* MTCC 5856 was added to 25 mL Aliquat 336 (50 % w/v in Oleyl alcohol) (Sigma Chemical Co., St Louis, MO, USA) and then stirred for 45 min. The mixture was centrifuged at 2000×*g* to separate the aqueous and organic layers. The organic layer was carefully transferred to a fresh tube. Similarly, glucose yeast extract broth was taken as blank control in the experiment. Optical rotation was measured using Automatic Polarimeter (AUTOPOL I, Rudolph Research Analytical, Hackettstown, NJ) by taking media control as the blank. Experiments were performed twice in triplicate.

### In vitro safety evaluation of *B. coagulans* MTCC 5856

#### Antibiotic resistance pattern

The minimum inhibitory concentrations (MICs) were determined as per the guidelines of Clinical and Laboratory Standards Institute (CLSI 2012). Briefly, the bacterial suspensions were prepared by suspending 18 h grown bacterial culture of *B. coagulans* MTCC 5856 in sterile normal saline (0.89 % NaCl w/v; HiMedia, Mumbai India). The turbidity of the bacterial suspension was adjusted to 0.5 McFarland standards (equivalent to 1.5 × 10^8^ colony forming units (cfu mL^−1^). Clindamycin, kanamycin, ampicillin, streptomycin, vancomycin, erythromycin, gentamicin, tetracycline and chloramphenicol were purchased from Sigma Chemical Co. (St Louis, MO, USA). The antibiotics stock solutions were prepared as per CLSI guidelines (CLSI 2012) and two fold serial dilutions were prepared in Mueller–Hinton broth (MHB, Difco Laboratories, Detroit, MI USA). The above mentioned bacterial suspension was further diluted in the MHB and 100 μL volume of this diluted inoculum was added to each well of 96-well U bottom microtiter plates (Tarson, Mumbai, India) resulting in the final inoculum of 5 × 10^5^ cfu mL^−1^ in the well and the final concentration of antibiotics ranged from 0.0078 to 4 μg mL^−1^. The plates were incubated at 37 °C for 24 h and were visually observed for the absence or presence of turbidity. The minimum concentration of the compound concentration showing no turbidity was recorded as MIC.

#### Bacterial reverse mutation assay

The mutagenic potential of *B. coagulans* MTCC 5856 by measuring the ability to induce reverse mutations at selected loci of *Salmonella typhimurium* in the presence and absence of rat liver S9 was performed as per Organization for Economic Cooperation and Development (OECD) guidelines (OECD 1997). Briefly, the tester strains of *S. typhimurium* histidine auxotrophs (TA98, TA100, TA102, TA1535 and TA1537) were received from Moltox Inc, Boone USA. The *B. coagulans* MTCC 5856, obtained at a concentration of 15 × 10^9^ cfu g^−1^, was mixed with sterile water not more than 30 min prior to use. Mutagenicity test was performed at concentrations of 312.5, 625, 1250, 2500, and 5000 µg plate^−1^, with and without the S9 mixture. 100 µL of tester strain (*S. typhimurium* strains), 50 µL of test article (*B. coagulans* MTCC 5856) were added to 2.0 mL of molten selective top agar at 45 ± 2 °C. After mixing thoroughly, the mixture was overlaid onto the surface of 25 mL of minimal glucose agar. The Petri plates were incubated for 48–72 h at 37 ± 2 °C. The plate incorporation methodology followed in this study was originally described by Ames et al. (1975) and updated by Maron and Ames (1983). Similarly, sterile water was used as the negative/vehicle control in the study. The positive control factors were methyl methane sulphonate (MMS), sodium azide, 9-aminoacridine (9-AA), Nitrofluorene and 2 aminoanthracene (2-AA). The experiment was performed both with and without an S9 activation system (Aroclor™ 1254-induced rat liver S9). Experiments were performed in triplicate.

#### Vero cell cytotoxicity assay

The ability of the *B. coagulans* MTCC 5856 to produce enterotoxins was determined by the method described by From et al. (2005). Briefly, a single isolated colony of *B. coagulans* MTCC 5856 and *Bacillus cereus* ATCC 14579 were added to brain heart infusion broth (HiMedia) supplemented with 1 % glucose and incubated at 32 °C for overnight. The optical density was checked at 600 nm and normalized to 1.0 for all the samples. 1 mL of this solution was further inoculated to 100 mL of fresh BHI broth supplemented with 1 % glucose (w/v) and incubated at 32 °C overnight. After incubation, samples were centrifuged at 16,100×*g* for 50 min and supernatants were stored at −20 °C until testing. Toxicity was determined by adding 100 µL of supernatant to cause swelling, rounding, and disseminating of the Vero cell layer. Each well was examined microscopically after 1, 3, and 5 h of incubation with 5 % CO_2_ and 37 °C and compared with a positive control supernatant (from *B. cereus* ATCC 14579 with the addition of 3, 10, 30, and 100 µL to four different wells) (From et al. 2005). After 5 h of incubation, media from the wells were removed and replaced with 100 μL of fresh media, 10 μL of 3-(4,5-dimethylthiazol-2-yl)-2,5-diphenyltetrazolium bromide (MTT) (Sigma) was added. Plates were further incubated for 3 h at 37 °C in a CO_2_ incubator. Formation of formazan salt by mitochondrial dehydrogenases was determined by an ELISA reader (Fluostar Optima, BMG LABTECH, Germany) at 565 nm. The percentage viability was calculated with respect to the untreated cells.

#### Detection of enterotoxin genes by PCR

The presence of *B. cereus*-like enterotoxin genes (*hblC, nheA, nheB, nheC* and *cytK*) in *B. coagulans* MTCC 5856 were detected by PCR amplification method as previously described (From et al. 2005). *B. cereus* ATCC 14579 was used as the positive control in the study. Briefly, *B. coagulans* MTCC 5856 and *B. cereus* ATCC 14579 were grown on sheep blood agar plates for 24 h at 30 °C. A loopful of all the cultures were inoculated into BHI broth supplemented with 1.0 % glucose and incubated at 30 °C for 6 h with shaking (120 rpm). After incubation, the cells were harvested by centrifugation and pellets were frozen at –20 °C overnight. Total DNA was extracted using lysozyme enzyme (10 mg mL^−1^) followed by the addition of DNAzol reagent (Thermo Fisher Scientific). PCR was performed by using a Gene Cycler (Bio-Rad). The primers for all five genes and PCR conditions were used as described by From et al. (2005). 16S rDNA gene as PCR positive control and primers without template were taken as negative control to validate PCR amplification conditions. Experiments were performed thrice independently at different experiments. PCR mixture (5 µL) was analyzed on a 2 % agarose gel.

#### Stability study of *B. coagulans* MTCC 5856

Stability of *B. coagulans* MTCC 5856 in powder form was studied to determine the shelf life at room temperature. Two standardized commercial preparations equivalent to 15 × 10^9^ and 6 × 10^9^ cfu g^−1^ were considered for the study. The long term stability study of *B. coagulans* MTCC 5856 was performed at room temperature according to ICH guidelines Q1A (R2) (ICH 2003). *B. coagulans* MTCC 5856 samples used in the study were manufactured by Sami Labs Limited (Bangalore, India) by following proprietary in-house manufacturing process (Majeed et al. 2016). Pure *B. coagulans* MTCC 5856 spores were spray–dried and diluted with maltodextrin (Sanwa Starch Co. Ltd. Kashihara, Nara, Japan) to achieve the desired concentration of 15 × 10^9^ and 6 × 10^9^ cfu g^−1^ for the finished product. Samples (50 g) were sealed inside double transparent polyethylene bags (4 × 4 in.) and then transferred to HDPE bottles capacity of 100 g which were stored at controlled temperature and humidity. Long term study was conducted at 25 ± 2 °C and relative humidity (RH) 60 % ± 5 % throughout the respective study period. Samples were analysed at 0, 3, 6, 9, 12, 18, 24 and 36 months. After every time interval, 1.0 g of *B. coagulans* MTCC 5856 sample was thoroughly mixed in sterile saline and then incubated in water bath for 30 min at 75 °C, followed by immediate cooling to below 45 °C. This suspension was further serially diluted in sterile saline and the viable count was enumerated by plating on glucose yeast extract agar (HiMedia, Mumbai, India) and then plates were incubated at 37 °C for 48–72 h. Each analysis was performed twice in triplicate. Average mean of spore viable counts are expressed in log_10_ cfu g^−1^.

### Statistical analysis

The values were calculated as the mean of individual experiments in triplicate and compared with those of the control groups. Differences between two mean values were calculated by Student’s *t* test. The chosen level of significance for all statistical tests was *P* < 0.05.

## Results

### Genetic consistency of *B. coagulans* MTCC 5856

#### Microscopy, colony morphology and physiological profiling

**Fig. 1:**
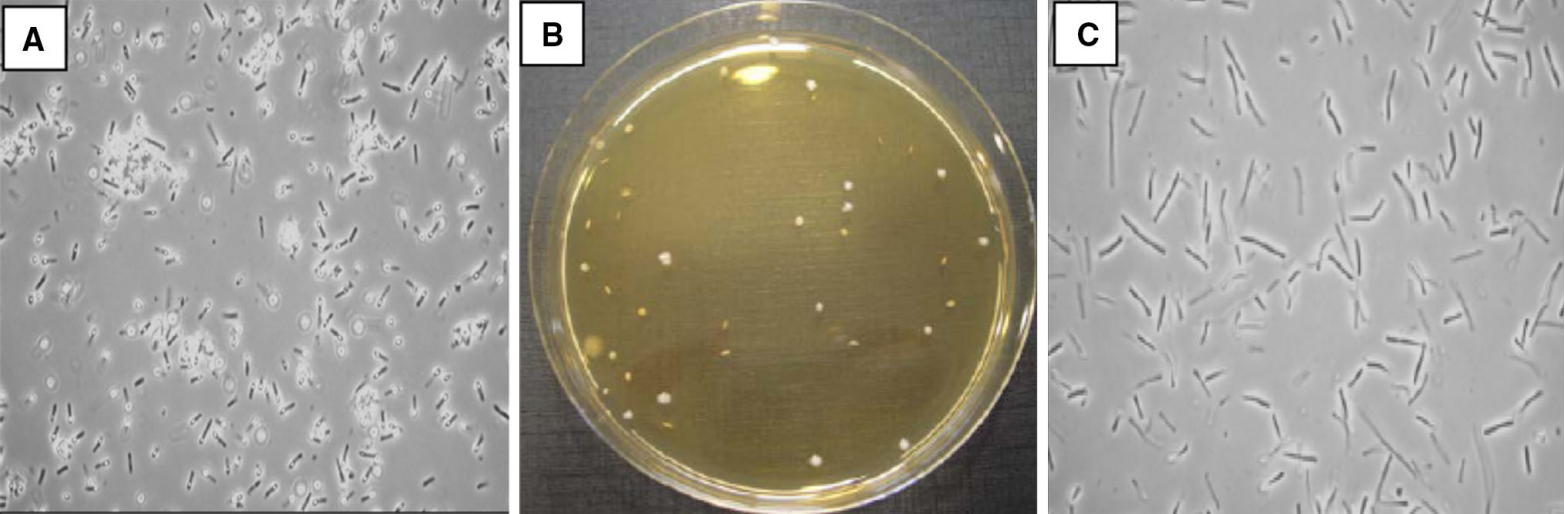
Phase contrast microscopic image (×1000 magnification) of *B. coagulans* MTCC 5856 powder (**a**), vegetative cells (**c**) and colony grown on GYE agar plate (**b**)

Samples from production lots of *B. coagulans* MTCC 5856 contain a mixture of ellipsoidal terminal spores and vegetative cells (Fig. 1a). Colonies from *B. coagulans* MTCC 5856 samples easily were grown on GYE media, yielding uniform, 1–3 mm in diameter, white to cream, smooth colonies (Fig. 1b) that contain vegetative rod shaped cells (Fig. 1b). Five different production lots were compared with reference sample for acid production from 21 sugars. The results showed that all colonies isolated from five different samples had an identical phenotype, consistent with the phenotype of *B. coagulans* (Table S1). Biochemical profiling and 16S rDNA confirmed that the strain present was *B. coagulans*, and that its identity was consistent over at least a period of 3 years.

#### GTG 5″ and BOX PCR fingerprinting

**Fig. 2:**
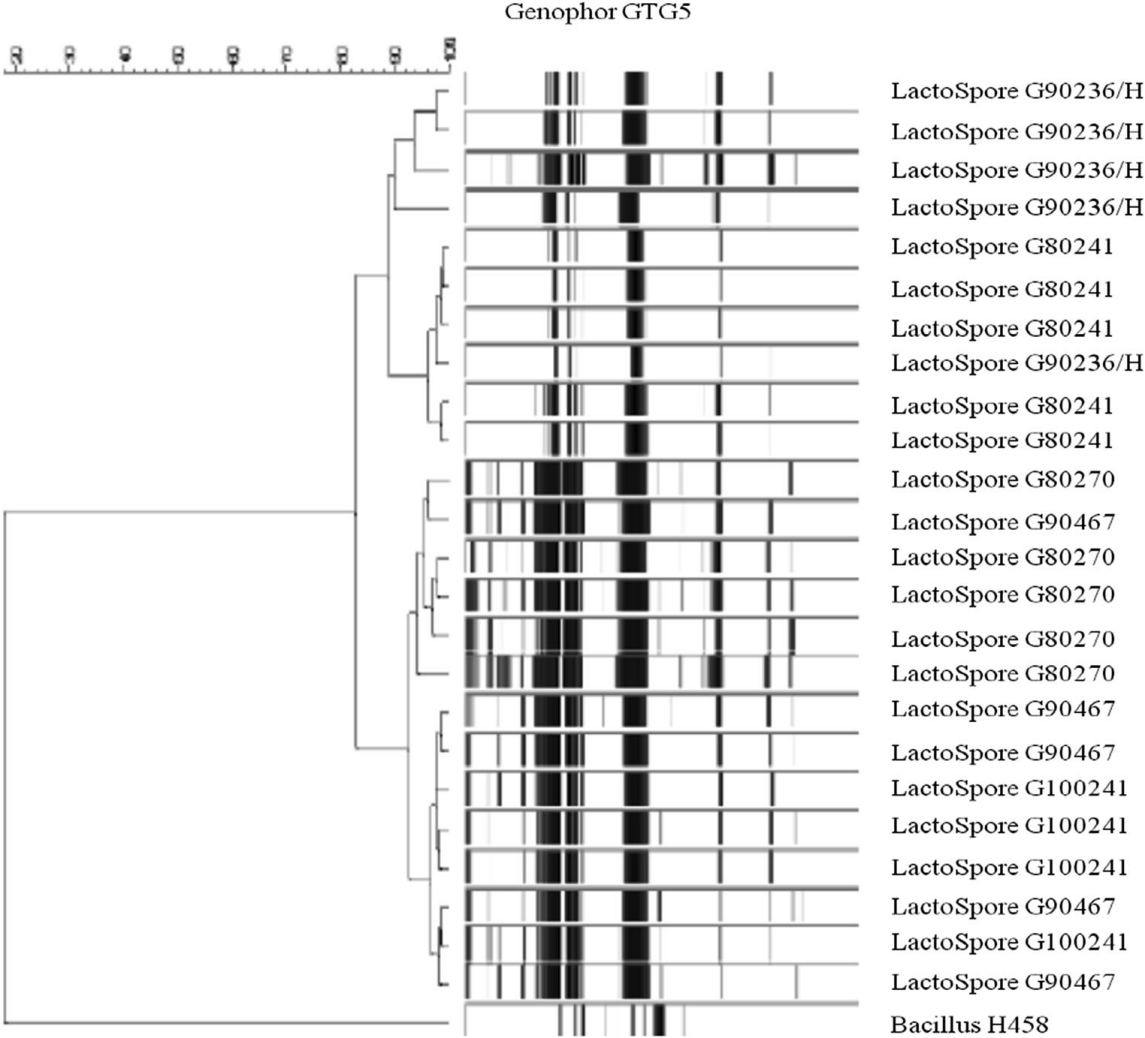
Cluster analysis of digitized banding patterns, generated by rep-PCR using the (GTG)5 primer, of *B. coagulans* MTCC 5856 isolates. The dendrogram was constructed using the unweighted pair-group method using arithmetic averages with correlation levels expressed as percentage values of the Pearson correlation coefficient. *Arrows* indicate the bands shared by the majority of the isolates

**Fig. 3:**
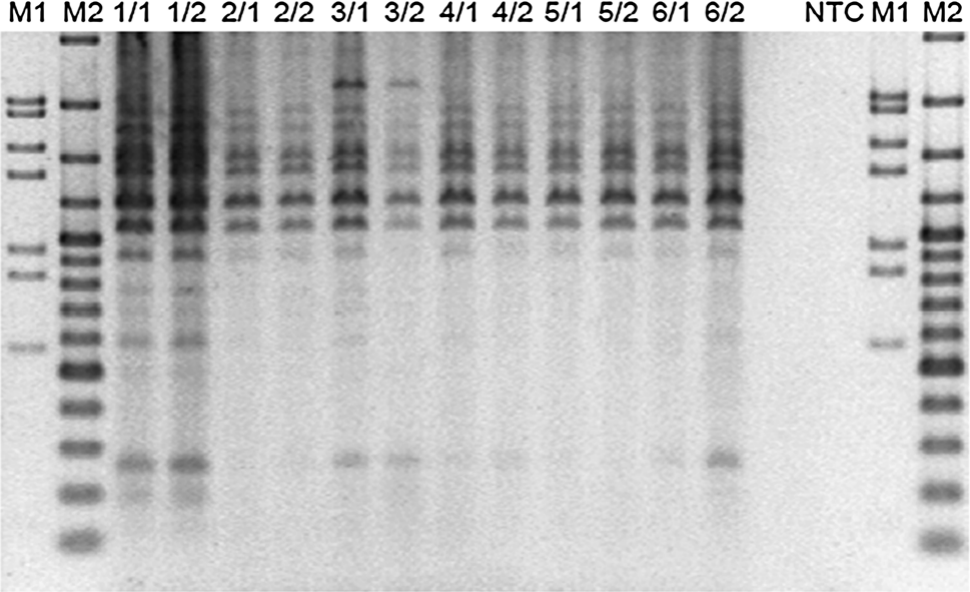
BOX-PCR fingerprints of the five *B. coagulans* MTCC 5856 samples. Two isolates form each sample was taken. Sample 1, LactoSpore^®^ lot G80241, sample 2, G90236/H; sample 3, original culture; sample 4, G80270, sample 5, G90467; sample 6, G100241

GTG 5″ fingerprinting analysis indicated that the sample contained one and the same strain over the 3 years period of production (Fig. 2). BOX-PCR fingerprints of the six *B. coagulans* MTCC 5856 samples (five production lots and one reference samples) were performed using repetitive intergenic DNA sequences (rep-PCR). Genotype of each strain could be distinguished according to distribution of PCR bands in different size. From each sample two independent colonies were tested. The isolated colonies from all five *B. coagulans* MTCC 5856 production samples had identical BOX PCR fingerprints (Fig. 3). This was another confirmation that the strain present was *B. coagulans*, and that its identity was consistent over at least 3 years of time.

#### Multi-Locus-Sequence typing

Multi-Locus-Sequence typing was performed on *B. coagulans* MTCC 5856 production batch samples targeting six household genes (*pta*, *tpi*, *glpF*, *ilvD*, *ldh*, and *pur*). Figure S1 shows the bands generated in the high fidelity PCR. The obtained sequences were aligned and compared to the sequences from an unrelated *B. coagulans* strain (strain 36D1), which was available in the public domain. The alignment of *ilvD* was shown as an example in Figure S2 (rest of the alignments are not shown). The alignments clearly showed that the sequences from the *B. coagulans* MTCC 5856 were all identical, but different at various points from 36D1, confirming the ability of the method to distinguish different strains from the same species. MLST study indicated that there was no change in the household genes, which are far more sensitive to mutation than the 16S rDNA gene. This was yet another confirmation of strain purity and consistency over a period.

### In vitro evaluation of probiotic potential of *B. coagulans* MTCC 5856

#### Resistance to gastric acid

**Fig. 4:**
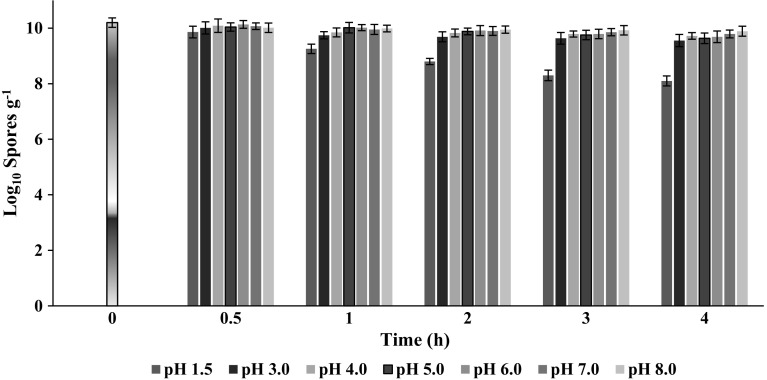
*Bacillus coagulans* MTCC 5856 survival at various pH values (1.5–8.0) in phosphate-buffered saline. The values are expressed in log_10_ spores g^−1^. Data represent the mean and standard deviations (±SD) of two different experiments performed in triplicate

There was no significant difference (4–7 %) in spore count at pH 3 to pH 8.0 in comparison to the initial spore count up to 4 h of the study (Fig. 4). However, 0.9 and 2.1 log_10_ reduction was observed at pH 1.5 in 1 and 4 h respectively. Results of the study confirmed the stability of *B. coagulans* MTCC 5856 spores in acidic as well as alkaline pH conditions.

#### Bile tolerance test

**Fig. 5:**
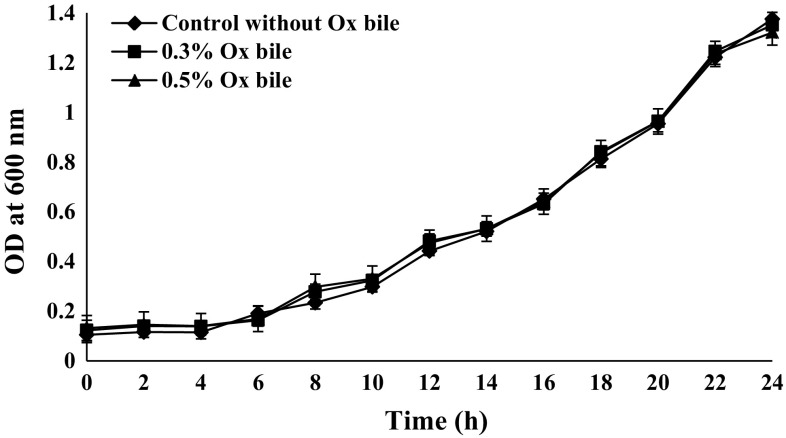
In-vitro effect of ox bile salt on the growth of *B. coagulans* MTCC 5856. The overnight grown fresh culture of *B. coagulans* MTCC 5856 was inoculated in MRS broth with and without 0.3 % ox bile salt (w/v) or 0.5 % ox bile salt (w/v). Values are mean (±SD) from three independent determinations. No significant difference of *B. coagulans* MTCC 5856 growth observed in presence and absence of ox bile (0.3 and 0.5 % w/v) (*P* > 0.05)

*Bacillus coagulans* MTCC 5856 growth was observed on the agar plate containing bile salt (1 % w/v) which indicated its tolerance against bile salt. Further, bile tolerance assay was performed by supplementing 0.3 and 0.5 % ox bile to the MRS broth. There was no significant difference of *B. coagulans* MTCC 5856 growth observed in presence and absence of ox bile (0.3 and 0.5 % w/v; Fig. 5). Similarly, there was no significant difference in the viability of *B. coagulans* MTCC 5856 in the presence and absence of bile salt (data not shown).

#### Antimicrobial activity against human pathogens

*Bacillus coagulans* MTCC 5856 showed broad spectrum antibacterial activity against human pathogens. It exhibited inhibitory activity against a panel of Gram positive and Gram negative pathogens including *M. luteus* NCIM 2169 (Table 1).

**Table 1:** Antimicrobial activity of *B. coagulans* MTCC 5856 against tested bacteria

S. no.	Tested organisms	Zone of inhibition (mm)
1	*Staphylococcus aureus* ATCC 29213	16.75 ± 1.0
2	*Staphylococcus epidermidis* ATCC 14990	14.00 ± 1.5
3	*Streptococcus mutans* MTCC 1943	17.75 ± 2.0
4	*Propionibacterium acnes* ATCC 11827	16.25 ± 0.6
5	*Bacillus cereus* ATCC 14579	16.75 ± 1.5
6	*Pseudomonas aeruginosa* ATCC 9027	18.75 ± 2.1
7	*Escherichia coli* ATCC 25922	19.50 ± 2.1
8	*Salmonella abony* NCIM 2257	16.00 ± 1.0
9	*Micrococcus luteus* NCIM 2169	17.00 ± 1.5

Anti-microbial activity was determined by well diffusion assay as described by Cintas et al. (1995). Data represent the mean ± SD of three independent experiments performed in triplicate

#### Production of lactic acid

Lactic acid production by *B. coagulans* MTCC 5856 was estimated by using a Megazyme kit. The total lactic acid produced by *B. coagulans* MTCC 5856 was 4.02 g/L. l-form of lactic acid was 3.99 g/L (>99 %) and d-form of lactic acid was 0.031 g/L. This was further confirmed by Polarimetric method and found l-form of lactic acid was exclusively produced by *B. coagulans* MTCC 5856.

### In vitro safety assessment of *B. coagulans* MTCC 5856

#### Antibiotic resistance pattern

The MIC values of antibiotics against *B. coagulans* MTCC 5856 are given in Table 2. All the tested antibiotics (clindamycin, kanamycin, ampicillin, streptomycin, vancomycin, erythromycin, gentamicin, tetracycline and chloramphenicol) showed a MIC range of 0.0078–1.0 μg mL^−1^ against *B. coagulans* MTCC 5856 indicating its susceptibility against antibiotics, meeting the requirements of EFSA.

**Table 2:** Minimum inhibitory concentrations (MICs) of antibiotics against *B. coagulans* MTCC 5856

S. no.	Antibiotics	MIC (μg mL^−1^)
1	Clindamycin hydrochloride	0.0078
2	Kanamycin sulphate	1.0
3	Ampicillin sodium salt	0.062
4	Streptomycin sulphate	1.0
5	Vancomycin hydrochloride	0.25
6	Erythromycin	0.125
7	Gentamicin sulphate	0.062
8	Tetracycline hydrochloride	0.062
9	Chloramphenicol	1.0

MICs of antibiotics were determined as per CLSI guidelines against *B. coagulans* MTCC 5856

#### Bacterial reverse mutation assay

The results of mutagenicity test show that *B. coagulans* MTCC 5856 spores did not increase the number of revertants in the five *Salmonella* strains (TA98, TA100, TA102, TA1535 and TA1537), compared with their negative controls, either absence or presence of the S9 metabolic activation system. Further, no dose-dependent mutagenic effects were caused by the *B. coagulans* MTCC 5856 spores (up to 5000 µg plate^−1^). *B. coagulans* MTCC 5856 spores did not show any mutagenic activity under the experimental conditions.

#### Vero cell cytotoxicity assay

**Fig. 6:**
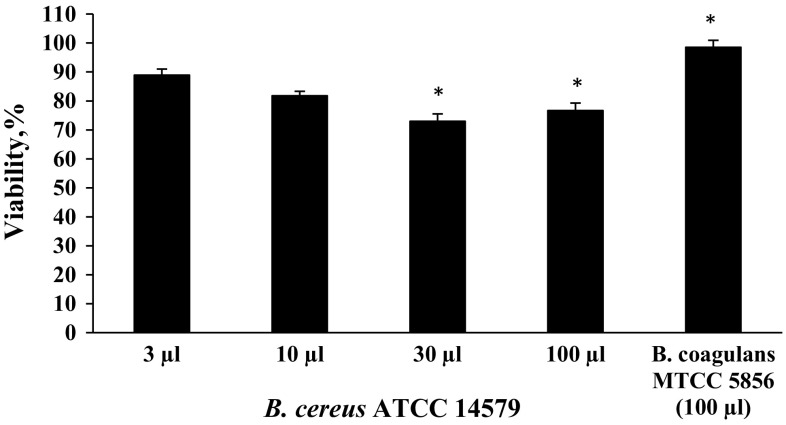
Effect of *B. coagulans* MTCC 5856 and *B. cereus* ATCC 14579 on the cell viability of Vero cell line. The percentage viability was calculated with respect to the OD of untreated cells (treated with brain heart infusion broth). Data represent the mean and standard deviations (±SD) of three different experiments performed in triplicate. *P < 0.05; (Student’s *t* test)

*Bacillus cereus* ATCC 14579 was taken as positive control in the study to compare with *B. coagulans* MTCC 5856. Microscopic observation revealed that no swelling, rounding and disseminating of Vero cells when incubated with 100 µL of supernatant of the *B. coagulans* MTCC 5856 for 5 h. However, *B. cereus* showed swelling, rounding and disseminating of Vero cells. Further, *B. cereus* ATCC 14579 supernatant (100 µL) showed 23 % reduction in viability of Vero cells when treated for 5 h. However, *B. coagulans* MTCC 5856 did not yield any PCR product, indicating the absence of *B. cereus*-like enterotoxin genes (Fig. 6).

#### Detection of enterotoxin genes by PCR

*Bacillus cereus* ATCC 14579 was found to be positive for all five *B. cereus* enterotoxin genes. However, *B. coagulans* MTCC 5856 did not yield any PCR product indicating absence of *B. cereus*-like enterotoxin genes (Fig S3).

#### Stability studies of *B. coagulans* MTCC 5856

**Fig. 7:**
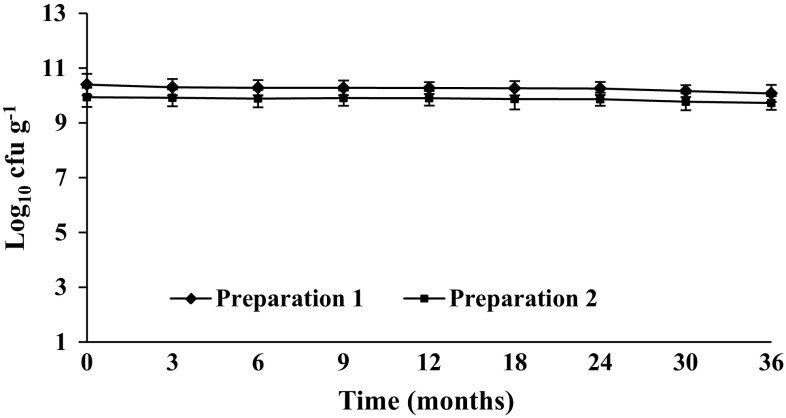
Viability of *B. coagulans* MTCC 5856 during the storage at room temperature (25 ± 2 °C with RH 60 % ± 5 %). Two standardized commercial preparations equivalent to 15 × 10^9^ (preparation 1) and 6 × 10^9^ cfu g^−1^ (preparation 1) were studied. Average means of spore viable counts are expressed in log_10_ cfu g^−1^. Each time point represents the mean log_10_ standard deviations (±SD) of three different experiments performed in duplicate

The results of *B. coagulans* MTCC 5856 stability studies are expressed in log_10_ cfu g^−1^ (Fig. 7). There were very negligible reductions in spore counts of *B.coagulans* MTCC 5856 in both the samples (15 × 10^9^ and 6 × 10^9^ cfu g^−1^) compared with the respective initial counts thus showing the stability of both the preparations over a period of 36 months (Fig. 7).

## Discussion

In this study, a detailed biochemical profiling data indicated that the same strain was present over three consecutive years of production. Further, random genomic technique GTG 5″ fingerprinting showed that all *B. coagulans* MTCC 5856 production batch samples had clustered together with at least 80 % similarity. This was an indication that all samples contained one and the same strain over the 3 years of production. Similarly, BOX PCR fingerprints were identical in all *B. coagulans* MTCC 5856 production samples. Random genomic fingerprinting techniques such as BOX–PCR fingerprint and GTG 5″ fingerprinting have been widely used for molecular typing of strains due to its good reproducibility, discriminative power, simple procedure, and low cost (Olive and Bean 1999; Zhu et al. 2006). This is the first detailed study to report the identity and genetic consistency of a commercial probiotic strain (*B. coagulans* MTCC 5856).

*Bacillus coagulans* is a very heterogeneous species with a high intra-species genomic diversity (De Clerck et al. 2004). The 16S rRNA sequence is suitable for estimating relatedness between different isolates, but cannot be used to discern exactly the different strains of *B. coagulans*. This means that strains with a nearly identical 16S rRNA sequence may have completely different economic, historical and practical traits (De Clerck et al. 2004). MLST data confirmed that there was no change in the household genes of *B. coagulans* MTCC 5856 strain over the period of 3 years unlike the other commercial probiotic preparations (Gilliland et al. 1984; Hughes and Hillier 1990; Canganella et al. 1997; Hamilton-Miller and Shah 2002; Weese 2002; Aureli et al. 2010). To the best of our knowledge, for the first time, the current study provides scientific evidence that the same strain of *B. coagulans* MTCC 5856 was present in multiple years of production of a commercial preparation.

The commercial probiotic strains may alter in different ways for a variety of purposes. The probiotic strain may undergo various phenotypic changes during the continuous and multiple years of commercial production (Sanders et al. 2014). These changes may alter the expression of physiological traits owing to the live nature of probiotics. Hence, genetic or phenotypic changes, by accident or design, might affect the efficacy or safety of commercial probiotics (Sanders et al. 2014). Therefore, we evaluated in vitro probiotic potential, safety and stability of the commercial probiotic strain (*B. coagulans* MTCC 5856), marketed as a dietary ingredient for nearly two decades. The in vitro study was conducted to mimic the condition of human gastrointestinal tract by treating the spores at highly acidic pH and growing *B. coagulans* MTCC 5856 at high bile salt concentration. *B. coagulans* MTCC 5856 showed resistance to highly acidic pH and high bile concentration. Probiotic organisms are reported to produce anti-microbial agents which inhibit the growth of pathogenic bacteria. Supernatant of *B. coagulans* MTCC 5856 exerted broad spectrum of anti-microbial activity by inhibiting not only the growth of Gram positive but Gram negative pathogens also. Lactic acid bacteria are known to produce two stereoisomers of lactic acid, namely, d and l lactic acid. l–lactic acid can be readily metabolized by human whereas the d-isomer cannot be metabolized and is considered to be a causative agent for acidosis (Coronado et al. 1995). The production of lactic acid by the *B. coagulans* MTCC 5856 was found to be l (+) lactic acid (>99 %) which was confirmed by Polarimetric method. This adds up to another property of *B. coagulans* MTCC 5856 for the safe use as probiotic product unlike recently reported complication by other probiotic products associated with d-lactic acid production (Munakata et al. 2010).

*Bacillus coagulans* MTCC 5856 was found to be susceptible to all the antibiotics recommended by European Food Safety Authority. The literature suggested that some antibiotic resistance genes can be transferred by probiotics such as *Lactobacillus reuteri* and *E. faecium* to the endogenous flora or to pathogens which may pose a health risk to human (Marteau 2001; EFSA 2012). Another safety concern associated with spore forming *Bacillus species* is the production of enterotoxins. *B. coagulans* MTCC 5856 did not cause any physiological or structural changes in Vero cell and had no significant cytotoxicity in MTT assay. Additionally, *B. coagulans* MTCC 5856 was found to be negative for *B. cereus*-like enterotoxin genes. Further, *B. coagulans* MTCC 5856 did not show any mutagenic effect in Ames test. The extensive in vitro safety studies revealed that *B. coagulans* MTCC 5856 is safe to use and thus eminently qualifies as a probiotic strain. It is essential to evaluate proper stability studies to identify probiotic contents and ensure that the organisms claimed on the label are actually present at the time the probiotic reaches a consumer. *B. coagulans* MTCC 5856 was found to be stable up to 36 months at ambient temperature confirming its shelf life for 3 years unlike the other probiotic products reportedly not having viable count matching the label claim (Canganella et al. 1997; Hamilton-Miller and Shah 2002; Weese 2002; Weese and Martin 2011). Majeed et al. (2016) reported that *B.* *coagulans* MTCC 5856 found to be stable during processing and respective storage conditions of baked food, beverages, vegetable oil, and concentrated glucose syrup and even in brewed coffee.

## Conclusions

In conclusion, *B. coagulans* MTCC 5856 (LactoSpore^®^) is a commercial probiotic preparation which contains *B. coagulans* as the active ingredient and the same strain was present over multiple years of production. The data presented in the current study suggests that the *B. coagulans* MTCC 5856 did not alter either genetically or phenotypically and was found to be consistent over multiple years of commercial production.

## Electronic supplementary material

Below is the link to the electronic supplementary material.

Supplementary material 1 (DOCX 504 kb)
