# Supplementary material for: Evaluation of genetic and phenotypic consistency of Bacillus coagulans MTCC 5856: a commercial probiotic strain
Source: World J Microbiol Biotechnol. 2016 Feb 29;32:60. doi: 10.1007/s11274-016-2027-2 (PMC4771827; doi:10.1007/s11274-016-2027-2)
Supplement: Supplementary file 1 — Supplementary material 1 (DOCX 504 kb) [file 11274_2016_2027_MOESM1_ESM.docx]

**Supplementary Data**

**Figure Legends**

**Fig. S1** Agarose gel (negative image) showing DNA fragments generated in the high fidelity PCR for MLST. Samples 7708, 7711, and 7720 were the production batches of *B. coagulans* MTCC 5856 and sample 7716 was the original culture.

**Fig. S2** Alignment of *ilvD* gene, showing the sequence from three different *B. coagulans* MTCC 5856 isolates and strain 36D1. Apart from the normal variability at the start and the end, the sequences from the *B. coagulans* MTCC 5856 samples were identical but at various points different from 36D1. Samples 7708, 7711, and 7720 were the production batches of *B. coagulans* MTCC 5856 and sample 7716 was the original culture.

**Fig. S3** Agarose gel (2%) showing the PCR amplification product of *B. cereus*–like enterotoxin genes in *B. coagulans* MTCC 5856 samples. All five enterotoxin genes (*hblC, nheA, nheB, nheC* and *cytK*) were found absent in the *B. coagulans* MTCC 5856. *Bacillus cereus* ATCC 14579 was used as positive control in the study. The experiment was performed thrice in two different occasions and results were found reproducible.

**Lane Description:**

**Lane L :** 100 bp ladder; **Lane P1,** PCR product of *B. cereus* ATCC 14579 with *hblC* primers; **Lane G1,** PCR product of *B. coagulans* MTCC 5856 with *hblC*  primers; **Lane N1,** PCR product without template with *hblC* primers; **Lane P2,** PCR product of *B cereus* ATCC 14579 with *nheA* primers; **Lane G2,** PCR product of *B. coagulans* MTCC 5856 with *nheA* primers; **Lane N2,** PCR product without template with *nheA* primers; **Lane P3,** PCR product of *B. cereus* ATCC 14579 with *nheB* primers; **Lane G3,** PCR product of *B. coagulans* MTCC 5856 with *nheB* primers; **Lane N3,** PCR product without template with *nhe B* primers; **Lane P4,** PCR product of *B. cereus* ATCC 14579 with *nheC* primers; **Lane G4,** PCR product of *B. coagulans* MTCC 5856 with *nheC* primers; **Lane N4,** PCR product without template with *nheC* primers; **Lane P5,** PCR product of *B. cereus* ATCC 14579 with *cytK* primers; **Lane G5,** PCR product of *B. coagulans* MTCC 5856 with *cytK* primers; **Lane N5,** PCR product without template with *cytK* primers; **Lane PP,** PCR Positive Control with 16S rRNA primers.

**Fig. S1**

**Fig. S2**

**Fig. S3**

L P1 G1 N1 P2 G2 N2 P3 G3 N3 P4 G4 N4 P5 G5 N5 PP L

**Table S1** Physiological test results of six *B. coagulans* MTCC 5856 samples. From each sample three independent colonies were tested, according to Kämpfer et al., (1991).

| Tests* | | G80241 | G90236 | REF | G80270 | G90467 | G100241 |
| --- | --- | --- | --- | --- | --- | --- | --- |
| Acid produced from: | |  |  |  |  |  |  |
|  | Glucose | +++ | +++ | +++ | +++ | +++ | +++ |
|  | Lactose | +++ | +++ | +++ | +++ | +++ | +++ |
|  | Sucrose | --- | --- | --- | --- | --- | --- |
|  | D-Mannitol | +++ | +++ | +++ | +++ | +++ | +++ |
|  | Dulcitol | --- | --- | --- | --- | --- | --- |
|  | Salicin | +++ | +++ | +++ | +++ | +++ | +++ |
|  | Adonitol | --- | --- | --- | --- | --- | --- |
|  | Inositol | --- | --- | --- | --- | --- | --- |
|  | Sorbitol | +++ | +++ | +++ | +++ | +++ | +++ |
|  | L-arabinose | +++ | +++ | +++ | +++ | +++ | +++ |
|  | Raffinose | --- | --- | --- | --- | --- | --- |
|  | Rhamnose | +++ | +++ | +++ | +++ | +++ | +++ |
|  | Maltose | +++ | +++ | +++ | +++ | +++ | +++ |
|  | D-Xylose | +++ | +++ | +++ | +++ | +++ | +++ |
|  | Trehalose | +++ | +++ | +++ | +++ | +++ | +++ |
|  | Cellobiose | +++ | +++ | +++ | +++ | +++ | +++ |
|  | Methyl-D-glucoside | +++ | +++ | +++ | +++ | +++ | +++ |
|  | Erythritol | --- | --- | --- | --- | --- | --- |
|  | Melibiose | --- | --- | --- | --- | --- | --- |
|  | D-Arabitol | --- | --- | --- | --- | --- | --- |
|  | D-Mannose | +++ | +++ | +++ | +++ | +++ | +++ |
|  |  |  |  |  |  |  |  |
| Hydrolysis of: | |  |  |  |  |  |  |
|  | Esculin | +++ | +++ | +++ | +++ | +++ | +++ |
|  | oNP-ß-D-galactopyranoside | +++ | +++ | +++ | +++ | +++ | +++ |
|  | pNP-ß-D-glucuronide | --- | --- | --- | --- | --- | --- |
|  | pNP-a-D-glucopyranoside | +++ | +++ | +++ | +++ | +++ | +++ |
|  | pNP-ß-D-glucopyranoside | +++ | +++ | +++ | +++ | +++ | +++ |
|  | pNP-ß-D-xylopyranoside | +++ | +++ | +++ | +++ | +++ | +++ |
|  | Bis-pNP-phosphate | +++ | +++ | +++ | +++ | +++ | +++ |
|  | pNP-phenyl-phosphonate | +++ | +++ | +++ | +++ | +++ | +++ |
|  | pNP-phosphoryl-choline | --- | --- | --- | --- | --- | --- |
|  | 2-Deoxythymidine-5'-pNP-phosphate | +++ | +++ | +++ | +++ | +++ | +++ |
|  | L-Alanine-pNA | --- | --- | --- | --- | --- | --- |
|  | L-Glutamate- γ-3-carboxy-pNA | --- | --- | --- | --- | --- | --- |
|  | L-Proline-pNA | --- | --- | --- | --- | --- | --- |
|  |  |  |  |  |  |  |  |
| Assimilation of: | |  |  |  |  |  |  |
|  | N-Acetyl-D-galactosamine | --- | --- | --- | --- | --- | --- |
|  | N-Acetyl-D-glucosamine | +++ | +++ | +++ | +++ | +++ | +++ |
|  | L-Arabinose | +++ | +++ | +++ | +++ | +++ | +++ |
|  | p-Arbutin | --- | --- | --- | --- | --- |  |
|  | D-Cellobiose | +++ | +++ | +++ | +++ | +++ | +++ |
|  | D-Fructose | +++ | +++ | +++ | +++ | +++ | +++ |
|  | D-Galactose | +++ | +++ | +++ | +++ | +++ | +++ |
|  | Gluconate | --- | --- | --- | --- | --- |  |
|  | D-Glucose | +++ | +++ | +++ | +++ | +++ | +++ |
|  | D-Mannose | +++ | +++ | +++ | +++ | +++ | +++ |
|  | D-Maltose | +++ | +++ | +++ | +++ | +++ | +++ |
|  | a-D-Melibiose | +++ | +++ | +++ | +++ | +++ | +++ |
|  | L-Rhamnose | +++ | +++ | +++ | +++ | +++ | +++ |
|  | D-Ribose | +++ | +++ | +++ | +++ | +++ | +++ |
|  | Sucrose | +++ | +++ | +++ | +++ | +++ | +++ |
|  | Salicin | +++ | +++ | +++ | +++ | +++ | +++ |
|  | D-Trehalose | +++ | +++ | +++ | +++ | +++ | +++ |
|  | D-Xylose | +++ | +++ | +++ | +++ | +++ | +++ |
|  | Adonitol | --- | --- | --- | --- | --- | --- |
|  | i-Inositol | --- | --- | --- | --- | --- | --- |
|  | Maltitol | --- | --- | --- | --- | --- | --- |
|  | D-Mannitol | +++ | +++ | +++ | +++ | +++ | +++ |
|  | D-Sorbitol | +++ | +++ | +++ | +++ | +++ | +++ |
|  | Putrescine | --- | --- | --- | --- | --- | --- |
|  | Acetate | --- | --- | --- | --- | --- | --- |
|  | Propionate | --- | --- | --- | --- | --- | --- |
|  | cis-Aconitate | --- | --- | --- | --- | --- | --- |
|  | trans-Aconitate | --- | --- | --- | --- | --- | --- |
|  | Adipate | --- | --- | --- | --- | --- | --- |
|  | 4-Aminobutyrate | --- | --- | --- | --- | --- | --- |
|  | Azelate | --- | --- | --- | --- | --- | --- |
|  | Citrate | --- | --- | --- | --- | --- | --- |
|  | Fumarate | --- | --- | --- | --- | --- | --- |
|  | Glutarate | --- | --- | --- | --- | --- | --- |
|  | DL-3-Hydroxybutyrate | --- | --- | --- | --- | --- | --- |
|  | Itaconate | --- | --- | --- | --- | --- | --- |
|  | DL-Lactate | --- | --- | --- | --- | --- | --- |
|  | L-Malate | --- | --- | --- | --- | --- | --- |
|  | Mesaconate | --- | --- | --- | --- | --- | --- |
|  | Oxoglutarate | --- | --- | --- | --- | --- | --- |
|  | Pyruvate | +++ | +++ | +++ | +++ | +++ | +++ |
|  | Suberate | --- | --- | --- | --- | --- | --- |
|  | L-Alanine | --- | --- | --- | --- | --- | --- |
|  | ß-Alanine | --- | --- | --- | --- | --- | --- |
|  | L-Aspartate | --- | --- | --- | --- | --- | --- |
|  | L-Histidine | --- | --- | --- | --- | --- | --- |
|  | L-Leucine | --- | --- | --- | --- | --- | --- |
|  | L-Ornithine | --- | --- | --- | --- | --- | --- |
|  | L-Phenylalanine | --- | --- | --- | --- | --- | --- |
|  | L-Proline | +++ | +++ | +++ | +++ | +++ | +++ |
|  | L-Serine | --- | --- | --- | --- | --- | --- |
|  | L-Tryptophan | --- | --- | --- | --- | --- | --- |
|  | 3-Hydroxybenzoate | --- | --- | --- | --- | --- | --- |
|  | 4-Hydroxybenzoate | --- | --- | --- | --- | --- | --- |
|  | Phenylacetate | --- | --- | --- | --- | --- | --- |

-, Negative reaction; +, positive reaction; oNP = ortho-nitrophenyl; pNP = para-nitrophenyl; pNA = paranitroanilide); REF = Reference sample; * The acid formation from different sugars and also the utilization of some carbon sources was sometimes weak. A slightly different behavior between the three samples in the development of the test results was visible.
